# Multivariate genetic architecture of poor sleep quality

**DOI:** 10.3389/fnins.2025.1647046

**Published:** 2026-01-14

**Authors:** Qihao Wang, Luqi Gao, Xiaoshan Yang, Bo Chen, Wenchen Li, Haifeng Wang

**Affiliations:** 1 Department of Neurosurgery, The Second Hospital of Jilin University, Changchun, China; 2 Department of Ophthalmology, The Second Hospital of Jilin University, Changchun, China

**Keywords:** genome-wide association study, genomic structural equation modeling, multivariate genetic architecture, nervous system disease, poor sleep quality

## Abstract

The field of genetics has yet to elucidate the complex genetic underpinnings that influence sleep quality. Previous studies have conducted genome-wide association studies (GWAS) on different dimensions of sleep health, but have not directly analyzed the multivariate genetic structure of poor sleep quality (PSQ). To address this knowledge gap, we employed a multifaceted approach that incorporated Genomic Structural Equation Modeling (Genomic-SEM) and multiple Post-GWAS methods. This strategy enabled us to identify causal single nucleotide polymorphisms (SNPs) that contribute to the variability in poor sleep quality. Our study identified a total of 14 leading SNP loci (such as rs2820309) and 3 fine-mapping significant loci (such as KTN1: rs77168063). To further investigate the underlying mechanisms, we employed multiple whole-transcriptome association methods. These methods analyzed susceptible gene signal loci that exhibited strong correlation with poor sleep quality, as determined by tissue, cell layer, and genome component analysis, along with related component information. Subsequently, data on approximately 13,000 common diseases were evaluated to determine the associated predisposing factors for poor sleep quality, and the correlation between poor sleep quality and 20 common neurological diseases was assessed. Additionally, we utilized a polygenic score based on summary data to analyze evidence of risk for poor sleep quality across different chromosomes. This study offers a novel perspective on the genetic underpinnings of poor sleep quality by conducting a genome-wide association study for a phenotype that was not directly measured.

## Introduction

Sleep quality is not only a tightly regulated mechanism; it is also a complex and repetitive biological process accompanied by changes in the nervous system (Paranhos et al., 2023). Genetics, environment, and lifestyle can profoundly influence sleep quality (Egan et al., 2017; Bruce et al., 2021). As people’s life pressures increase, the incidence of poor sleep quality is rapidly increasing (Lašaitė and Radzevičienė, 2024; Gao et al., 2018), and it and its relationship with neurological diseases are becoming major challenges in the medical and socioeconomic fields (Brandt, 2021). Notwithstanding the noteworthy advancements in the field of sleep research in recent years, the specific genetic and biological underpinnings of poor sleep quality (PSQ) remain to be fully elucidated (Buysse, 2013; Zhang and Fu, 2020). Previous studies have discussed the genetic correlates of different dimensions of sleep health, but have not directly addressed the underlying genetic architecture of PSQ (Morrison et al., 2024). While studies have demonstrated that PSQ may be a significant contributor to neurological diseases, these findings are insufficient to fully account for the variability in sleep quality among individuals and the differences in disease susceptibility (Khatami, 2014; Mayer, 2016). To address these challenges, the present study aims to explore potential molecular mechanisms and expand and diversify the connections to multiple potential diseases by integrating multiple genetic analysis tools and strong correlation exploration tools. In particular, the present study focuses on multiple genomic loci and chromosomal regions associated with PSQ to reveal potential possibilities for improving sleep quality. This study not only expands our understanding of sleep quality, but also provides theoretical and practical support for sleep management and intervention strategies related to neurological diseases.

In order to address the current lack of precise measurements of the mechanisms underlying PSQ, a GWAS study of potential unmeasured sleep quality was designed. Genomic structural equation modeling (Genomic-SEM; Grotzinger et al., 2019) was applied to published GWAS summary statistics for diseases and biomarkers associated with PSQ. Utilizing these statistics, we obtained the associations of these SNPs with the latent poor sleep phenotype, thereby establishing a GWAS study of the latent PSQ phenotype that has never been directly measured. Furthermore, we employed comprehensive analysis methods in systems biology to define the portion of genetic variation in PSQ that is not explained by known biomarkers as potentially relevant genetic markers. We conducted various GWAS-related studies on them. Despite its limitations in fully capturing the intricate interplay between sleep quality pathways and multifactor interactions, this approach offers a distinct advantage by circumventing the confounding effects of sleep quality markers, facilitating the analysis of otherwise challenging-to-study data (Yang et al., 2010). Finally, from a direct application perspective, we performed multiple correlation analyses to construct a simple influence factor map of PSQ and neurological diseases for non-biostatisticians (clinicians, etc.). The purpose is to enable non-biostatisticians to directly apply the relevant influence factor map to develop potential preventive and intervention measures for patients. Our research aims to create an easy way from genomic statistics to basic research and clinical measures.

## Methods

### Single input GWAS data sources

Our individually variable input GWAS data were obtained from 6 GWAS involving aspects related to poor sleep quality, including Trouble falling asleep (TFA), Insomnia (Ins), Undersleep (Und), Sleep disorders (SlD), Hypnotic drug dependence (HDD), and Tiredness (Tir). All input GWAS had ethical clearance from their respective institutional review boards, and all participants provided informed consent, and the data were subjected to strict quality control. Of these, TFA (*n* = 243,876) was from a study by Verma et al. (2024), Und (*n* = 110,188) was from a study by Jones et al. (2016), HDD (*n* = 146,106) from a study by Jiang et al. (2021), Ins (*n* = 497,539) and SlD (*n* = 495,270) from Finngen: https://www.Finngen.fi/en (Kurki et al., 2023), Tir (*n* = 449,019) from the IEU OpenGWAS project: https://gwas.mrcieu.ac.uk (Lyon et al., 2021; detailed GWAS listing information is available in Supplementary Table 1).

### Quality control for single-input GWAS

Firstly, it is imperative to exclude low-quality samples, defined as those with a missing rate exceeding 5%. Next, the MHC region (MHC, major histocompatibility complex) is located on chromosome 6, at a specific location between approximately 25,000,000 and 35,000,000 base pairs (genomic position). Due to the genetic diversity and structural complexity of the MHC region, especially the polymorphism of immune-related genes (Plasil et al., 2019; Lie and Thorsby, 2005), the MHC region is usually specially processed. Subsequently, the construction of a GWAS was initiated. In this study, the default parameters were utilized during the preparation of summary statistics. The autosomal SNPs from the five input PSQ GWAS that passed the recommended default quality control filters were filtered to the 1,000 Genomes Phase 3 EUR panel. SNPs with a minor allele frequency (MAF) < 0.01 were removed. These SNPs are error-prone due to the small number of samples in the genotype cluster, and the standard error of the regression of the linkage disequilibrium (LD) score for these SNPs is usually high (Lachance, 2010). SNPs with zero effect estimate were removed (to avoid affecting matrix reactivity, which is necessary for the Genomic-SEM), SNPs that did not match the reference panel were removed, palindromic SNPs with uncertain direction were excluded, strand inversions were corrected by referencing a unified allele coding scheme, and SNPs with mismatched alleles were excluded.

### Sample overlap in single-input GWAS

In our analysis, the single-input GWAS we included came from different genome repositories and had different participants. This means that when conducting the GWAS, we fully considered the sample overlap between different cohorts to ensure the accuracy and completeness of the results, as well as the statistical impact of potential sample overlap.

### Genomic structural equation modeling

**Figure 1:**
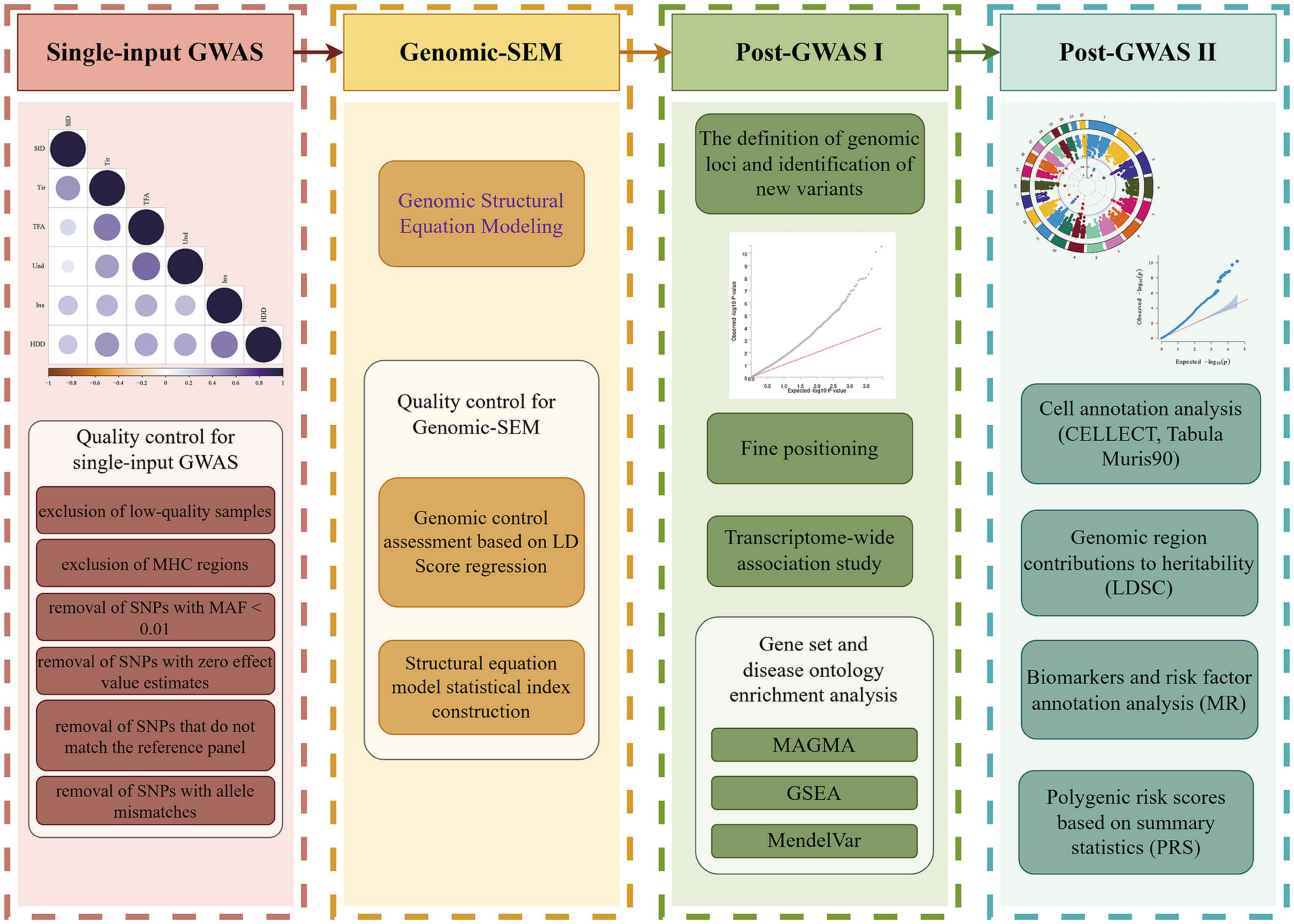
Flowchart for genomic structure equation modeling and subsequent analysis (which include linkage disequilibrium score regression analysis of 6 single input GWAS data; Q-Q plot of the gene-based test computed by MAGMA; circular Manhattan diagram from the TWAS and Q-Q plot from the TWAS). TFA, Trouble falling asleep; Ins, Insomnia; Und, Undersleep; SlD, Sleep disorders; HDD, Hypnotic drug dependence; Tir, Tiredness; GWAS, Genome-wide association study; MAGMA, Multi-marker analysis of genomic annotation; TWAS, Transcriptome-wide association study (by Figdraw).

We used the GenomicSEM R package (v.0.0.5) to implement genomic structural equation modeling of TFA, Ins, Und, SlD, HDD, and Tir in a GWAS analysis to investigate the broad genetic susceptibility behind these PSQ related traits (Figure 1, By Figdraw). Genomic-SEM is a newly developed multivariate approach that can examine multiple latent multivariate models to explore the potential structure of traits of interest (Grotzinger et al., 2019; refer to Table 1 for detailed criteria.).

**Table 1:** Detailed parameters of each GWAS data in the structural equation modeling.

Phenotype	NSNPs	h^2^ (se)	λGC	Mean ChiSquare	Intercept (se)	Ratio (se)
TFA	1,172,607	0.0456 (0.0029)	1.2694	1.3056	1.0857 (0.0074)	0.2803 (0.0241)
Ins	1,160,400	0.0056 (0.001)	1.0648	1.0667	1.0113 (0.0073)	0.1689 (0.1088)
Und	1,152,859	0.0484 (0.0046)	1.0966	1.1091	1.0062 (0.0059)	0.057 (0.0543)
SlD	1,160,405	0.0421 (0.0021)	1.4529	1.5224	1.1098 (0.0112)	0.2102 (0.0215)
HDD	1,154,566	0.0047 (0.0033)	1.0086	1.0026	0.9889 (0.0056)	4.2737 (2.1765)
Tir	1,175,018	0.0596 (0.0025)	1.4295	1.5668	1.0379 (0.0089)	0.0668 (0.0158)

GWAS, Genome-Wide Association Studies; TFA, Trouble falling asleep; Ins, Insomnia; Und, Undersleep; SlD, Sleep disorders; HDD, Hypnotic drug dependence; Tir, Tiredness.

Genomic-SEM is not biased by sample overlap (e.g., UKB participants overlapping in multiple input GWAS validation) or sample size imbalance. Furthermore, this method facilitates the identification of variants that affect only some but not all complex traits, which do not represent a broad cross-trait susceptibility.

Genomic-SEM is a two-stage process (Nock and Zhang, 2011). In the first stage, the empirical genetic covariance matrix and the corresponding sampling covariance matrix are estimated. We prepared summary statistics for the PSQ GWAS for the first stage and used a multivariate extension of cross-trait LDSC (Linkage disequilibrium score regression; Bulik-Sullivan et al., 2015) to generate the empirical genetic covariance matrix between the six traits as input for the SEM common factor model. In the subsequent stage, an SEM model was specified with the objective of minimizing the discrepancy between the assumed and the empirically calculated covariance matrices from the initial stage. The primary research objective was to identify the genetic underpinnings of the six sleep quality-related traits. To that end, a univariate model was tested. The model fit was evaluated using SRMR, model χ^2^, the Akaike information criterion, and CFI (comparative fit index). By implementing the appropriate common-factor SEM specification, individual autosomal SNPs were incorporated into the genetic and related sample covariance matrices, thereby yielding a polygenic broad-sense heritability result of 5,761,413 shared covariance between related GWAS.

### Genomic structural equations SNP heterogeneity

To assess whether SNP associations are appropriately modeled within a multivariate structural equation modeling (SEM) framework, we computed the SNP heterogeneity statistic. The original hypothesis of the SNP test was that SNP associations in a single phenotypic GWAS are statistically moderated by a constructed GWAS for PSQ. Thus, significant QSNP testing in the constructed GWAS suggests that SNPs may be associated with pathways other than the shared genetic mechanisms in the constructed model. SNPs with significance thresholds (*p* < 0.05) were subsequently excluded.

### The definition of genomic loci and identification of new variants

The FUMA GWAS (Functional Mapping and Annotation of Genome-Wide Association Studies) method was utilized to identify genomic loci and to ascertain the leading SNP loci associated with the constructed GWAS (Watanabe et al., 2017; Watanabe et al., 2019). These SNPs exhibited a low correlation (less than 0.1) with other SNPs in LD and possessed genome-wide significance (*p* value < 5 × 10^−8^). Initially, the summary statistics of the SNPs from the constructed GWAS were entered into the program to assess their association strength. Furthermore, a comparison was made between the leading SNP loci and the original single-input GWAS. To ascertain whether the 14 leading SNP loci in the new GWAS were associated with multiple effects, the GWAS Catalog was consulted for published significant associations (*p* value < 5 × 10^−8^). Furthermore, a risk gene locus analysis was performed on the established model based on the fuma software function, with a significance threshold of *p* value < 5 × 10^−8^, and the relevant output file was analyzed with MAGMA (Multi-marker Analysis of GenoMic Annotation). MAGMA is a tool for post-processing GWAS that aims to assess associations between genes and phenotypes such as disease or health traits. The MAGMA tool integrates multiple genetic markers (e.g., SNPs) into a gene-level signal, and calculates the association of each gene with the phenotype. The objective is to extract information about gene function from genome-wide SNP data in order to analyze genetic signals at the gene level, with a significance threshold of FDR-*p* value < 0.05. Furthermore, a novel methodology has been developed, which is referred to as the GWAS locus reduction method. This method compares the genome-wide significance threshold used in single-input GWAS with the lead sites identified by Genomic-SEM. The implementation of this method has the potential to facilitate the identification of additional valuable novel sites at the lead site.

### SuSIE and FINEMAP

In order to identify the most likely causal variants associated with the new GWAS, SuSIE (Sum of Single Effects) and FINEMAP were utilized, with the latter being implemented in the R package echolocatoR v.2.0.3 (Akdeniz et al., 2024). Identifying possible causal variants using SuSIE and FINEMAP: SuSIE and FINEMAP are both tools for fine-mapping analysis, which aim to identify the most likely causal variants associated with a phenotype. In this step, a 250 kb window was used to include the regions associated with each lead SNP, and the causal inference probability of each SNP within these regions was calculated. Confident set: A probability threshold of 0.95 was set. If the posterior probability of a variant exceeds this threshold, it is designated as a possible causal variant. Consensus SNP and probability set: echolocatoR defines a ‘consensus SNP’, that is, a variant that appears in both SuSIE and FINEMAP results. For these consensus SNPs, the tool calculates their average posterior probability and determines the average credible set based on the probability results. The credibility is defined as 1 when the posterior probability of the SNP in SuSIE and FINEMAP exceeds 0.95, otherwise it is 0.

### Whole genome association study

Following the localisation of possible causal variants, a TWAS (Transcriptome-Wide Association Study) was performed to prioritize genes associated with the constructed GWAS based on the relationship between gene expression and phenotype (Mai et al., 2023; Li et al., 2021). The FUSION method was used for TWAS, and 37,920 pre-computed expression quantitative trait loci (eQTL) traits from GTExv.8 data were utilized. These were then used to calculate the expression associations between different genes and tissues.

A subsequent analysis of the TWAS results revealed that the new GWAS data contained sufficient variation to analyze 36,149 traits (from 37,920 eQTL traits), thereby indicating the data’s high quality. Genes with a *p* value of less than 0.05 (genes significantly associated with the constructed SEM) were included in further analysis. For these TWAS significant genes, the FOCUS method (a fine-mapping method designed specifically for TWAS studies) was further performed, and the FOCUS method was used to prioritize novel GWAS genes. The FOCUS method assesses whether there is a causal relationship between a gene and a phenotype based on the FOCUS posterior inclusion probability. Combining with previous studies, we considered TWAS significant genes that not only showed significance in the TWAS analysis, but also were consistent with other evidence (such as FOCUS), indicating that they may be causal.

### Gene set and disease ontology enrichment analysis

In order to investigate the potential relationship between PSQ and Mendelian disease genes and their related pathways, MAGMA and FUMA (GESA) data were used for gene enrichment analysis and gene pathway set analysis. In addition, MendelVar^1^ was used for gene enrichment analysis (Sobczyk et al., 2021).

### Cell annotation analysis

In order to identify the cell types associated with PSQ, Cell Type Expression Specificity Integration for Single-Cell RNA Sequencing Data of Complex Traits (CELLECT) was utilized (Timshel et al., 2020), with the Tabula Muris90 dataset being employed as a source of data (Tabula Muris Consortium, Overall coordination, Logistical coordination, Organ collection and processing, Library preparation and sequencing, Computational data analysis, Cell type annotation, 2018). This particular dataset contains transcriptome data from 100,000 cells and 20 organs and tissues from mice (*Mus musculus*). The CELLEX was then utilized for the preprocessing and normalization of the single-cell RNA sequencing data from Tabula Muris, followed by the calculation of the expression-specific likelihood score for each gene. Subsequently, the LDSC software was employed for cell type-specific analysis, and the cell types were classified.

### Genomic region contributions to heritability

The LDSC tool is utilized to compute partitioning heritability, with the contribution of each genomic region to heritability being assessed by assigning the genetic information of a phenotype to different genomic regions (genes, enhancers, silencers, etc.). Specifically, LDSC employs a weighted LD matrix, a genotype frequency file, and summary statistics to perform the calculation. This process ultimately provides an estimation of the genetic contribution of each region.

### Biomarkers and risk factor annotation analysis

In order to identify the degree of association between previously measured diseases and biomarkers and the constructed GWAS data on PSQ that was not directly measured, a large-scale correlation analysis was performed. The analysis incorporated 13,014 phenotypes from the IEU database as potential exposure factors and 20 common phenotypes of neurological diseases from the IEU and FinnGen databases as potential outcome factors. Mendelian randomization (MR) analysis was performed using the TwoSampleMR package v.0.6.8, with inverse variance weighted (IVW) as the primary correlation test (*p* < 0.05). The final results underwent Bonferroni multiple correction.

### Construction of polygenic risk scores based on summary statistics

Polygenic risk scores (PRS; Ge et al., 2019) were calculated based on genome-wide summary statistics, and the genetic contribution of different chromosomal regions to the development of the disease was assessed. The method utilized PRS-CS (Polygenic Risk Score with Continuous Shrinkage) software to estimate the posterior effect values of SNPs through GWAS data and an external LD reference panel. PRS-CS was implemented using the standard European LD reference panel from the 1,000 Genomes Project, and default PRS-CS parameters were used. PRS is calculated using a Bayesian regression model that integrates an LD reference panel based on GWAS summary statistics.

## Results

### Structural equation model statistical index construction

LD-Score regression analysis of the six univariate inputs into the GWAS for TFA, Ins, Und, SlD, HDD, and Tir had heritability contributions Z-values of 15.9, 5.49, 10.5, 20.1, 1.44, and 23.8, respectively. The Z-values of the genetic covariates for their two bivariate inputs were 10.8 (TFA and Und), 16.2 (TFA and Tir), 2.31 (Ins and HDD), and detailed one-way genetic parameters are detailed in Supplementary Table 2. The six genetic covariance matrices fitted well to the common factor model, as indicated by the following fit indices: comparative fit index (CFI) = 0.93 and standardized root mean square residual (SRMR) = 0.09. A detailed assessment of the model stability is provided in Supplementary Table 3. The univariate SEM parameters for the potential factors (F1) are presented in Supplementary Table 4. These results collectively suggest evidence of shared genetic factors.

### Genomic structural equation modeling

By extending SEM to incorporate individual variation, we generated an indirectly measured obtained GWAS estimating the association of 5,761,413 SNPs with PSQ.

### Genome structural equation model based on LD score regression genome control assessment

We have removed a total of 4,777,855 SNPs through parameter control in the method, and a total of 983,558 effective SNPs have been retained after retaining the regression coefficients. The Mean chi^2^ value for all SNPs is 1.45, the genome control Lambda GC is 1.398, extreme value standard Max Chi^2^ is 40.019, genome-wide significance level is 78, total observed scale heritability (h^2^) is 0.0038(0.0002), genetic contribution to environmental contribution Ratio is 0.0178(0.0214), intercept term in the regression model is 1.008, standard error of the intercept term in the regression model is 0.0096. The multiple estimates directly indicate that the potential inflation of the structural equation we are concerned with is due to the polygenic heritability signal, rather than population stratification bias, and pleiotropic parameter effects.

### Structural equation model evaluation based on FUMA software

**Figure 2:**
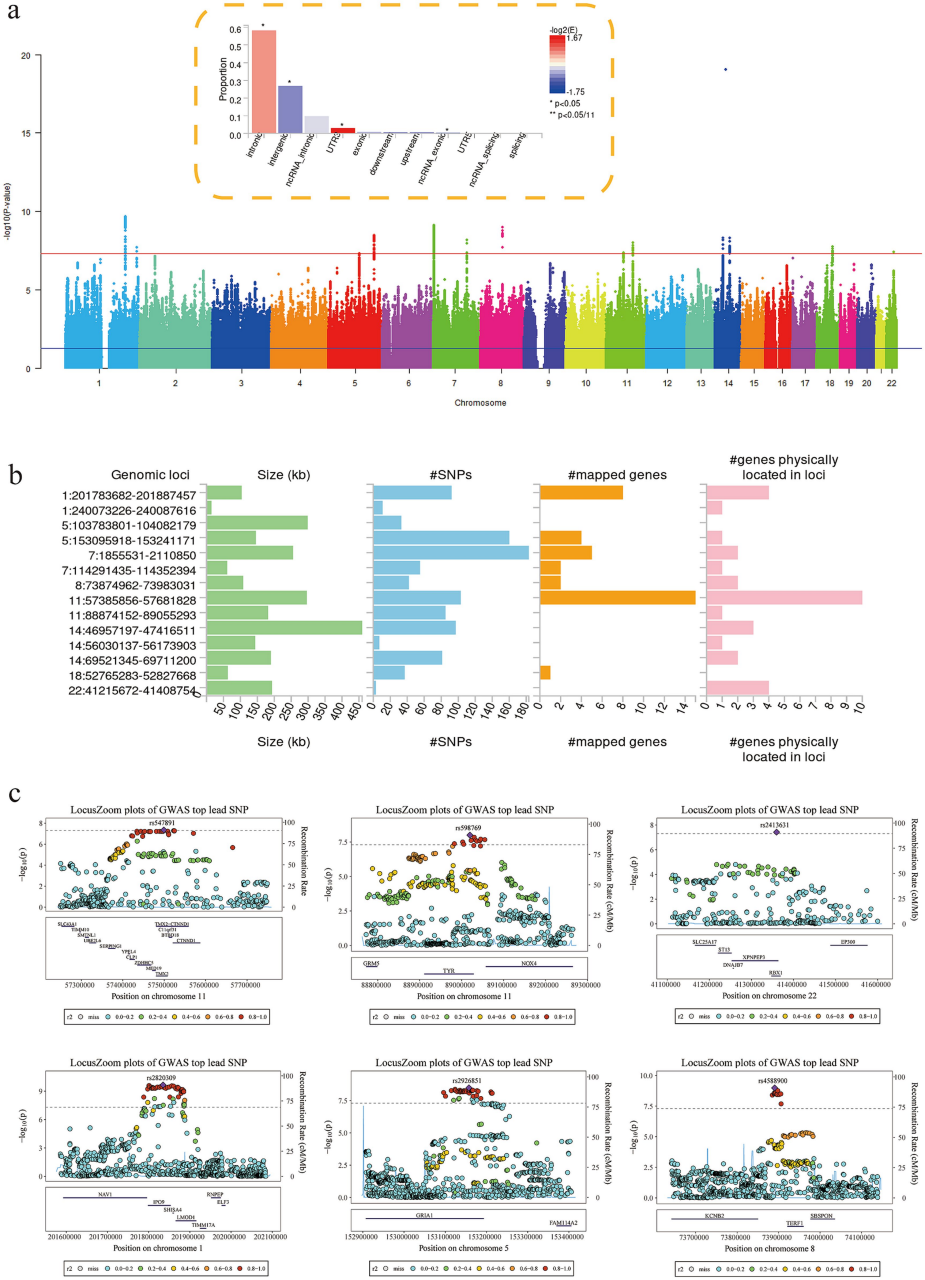
Functional mapping and annotation of GWAS. **(a)** Manhattan plot of GWAS summary statistics (only SNPs with *p*-value ≤ 1 × 10^−5^ are kept); Functional consequences of SNPs on genes. **(b)** Summary per genomic risk locus. **(c)** Leading SNP loci detected through FUMA (rs547891, rs598769, rs2413631, rs2820309, rs2926851, rs4588900). GWAS, Genome-ide ssociation tudy; SNPs, single nucleotide polymorphisms.

Using FUMA software (Figures 2a,b), we evaluated the Genomic-SEM and found 14 leading SNP loci (Figure 2c; Supplementary Table 5a). We also identified a total of 460 potential genes associated with PSQ through genome-wide significant control (Significance was defined as a genome-wide *p* < 5 × 10^−8^; findings with FDR < 0.05 were considered suggestive; Supplementary Table 5b). The vast majority of these 14 leading SNP loci are located in intronic and intergenic (Supplementary Table 6). In addition, a total of 8 GWAS reduction loci (including more valuable novel loci identified among leading SNP loci) were identified (such as rs2820309, rs6421926, rs4588900, etc.; Supplementary Table 7). rs6421926 has been reported in multiple research papers (Akimova et al., 2025; Baselmans et al., 2019), but we found that this site is not directly related to PSQ. Instead, it is a potential mediator site. rs4588900 has been reported in previous studies of European populations to be associated with snoring (Watanabe et al., 2022; Jansen et al., 2019), which is relevant to the phenotypes in our study.

### Fine mapping

**Figure 3:**
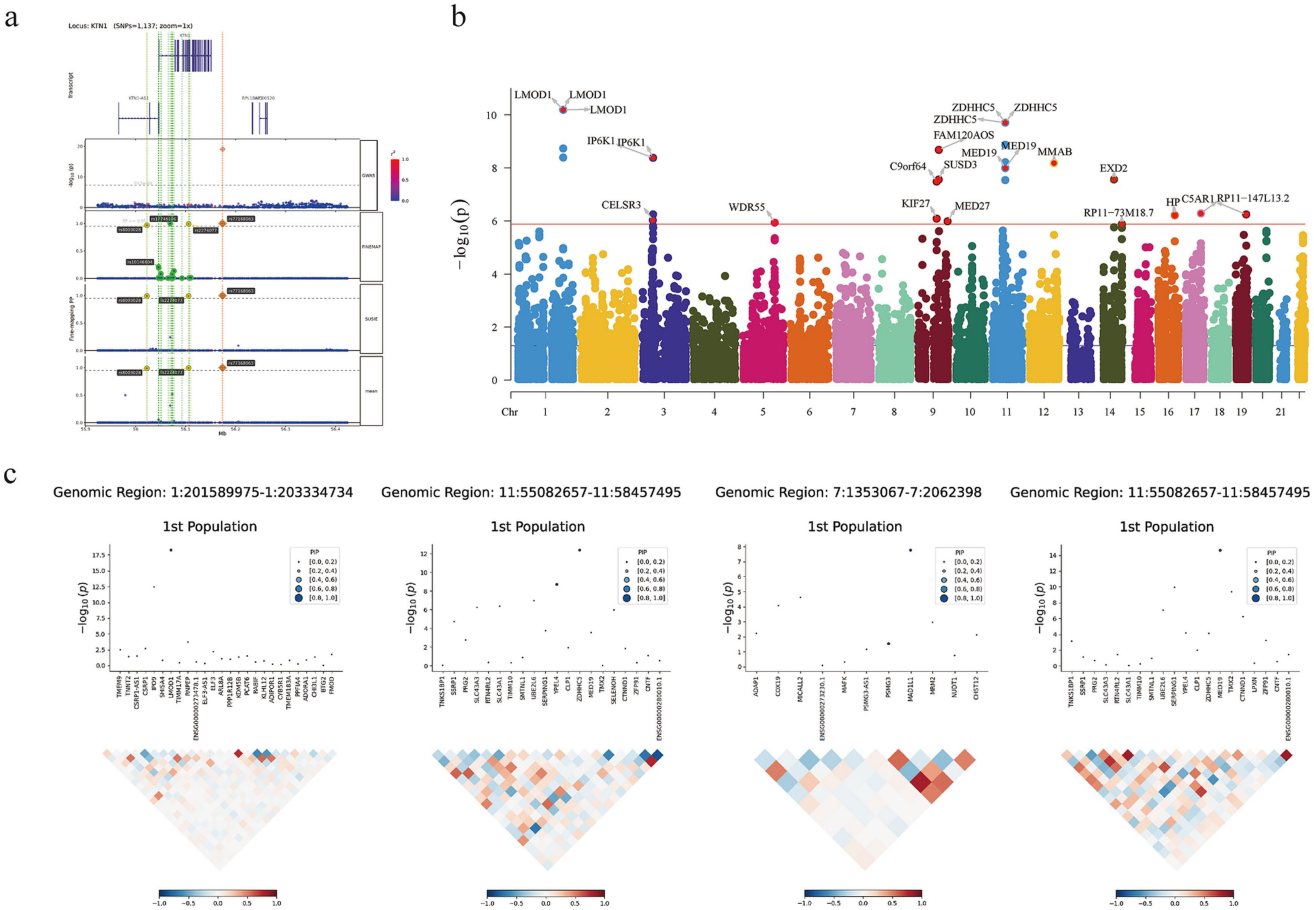
Fine mapping and TWAS. **(a)** Fine localization analysis identified strong associations at multiple genomic locations (mean. PP > 0.95; KTN1). **(b)** Manhattan plot of 23 genes that exceeded the criteria for correction for multiple comparisons from the TWAS. **(c)** FOCUS fine positioning analysis results (LMOD1, ZDHHC5, MAD1L1, MED19). TWAS, Transcriptome-Wide Association Study.

Fine Mapping Analysis identified strong associations at multiple genomic loci (mean. PP > 0.95), including: chromosome 14 (rs8003028, rs2274077, rs77168063, variant in KTN1). Regional plots showed significant peaks at these loci, and other credible set variants also showed evidence of association (Figure 3a; Supplementary Table 8).

### Transcriptome-wide association study

**Figure 4:**
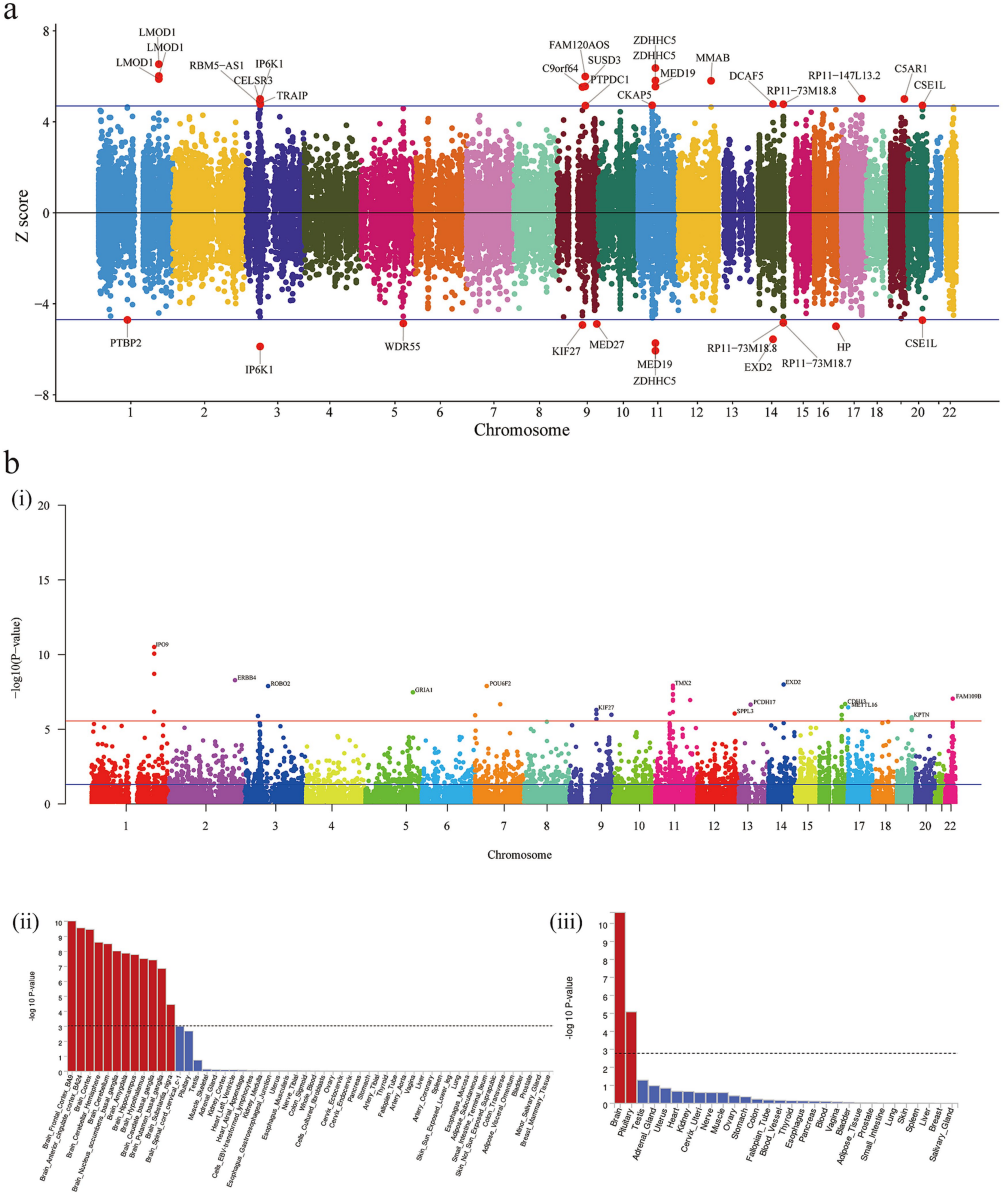
TWAS and MAGMA. **(a)** Manhattan plot of *Z*-scores from the TWAS. **(b)** (i) Manhattan plot of the gene-based test as computed by MAGMA based on GWAS summary statistics. Genome wide significance (red dashed line in the plot) was defined at *p* = 0.05/18210 = 2.746 × 10^−6^; (ii,iii) MAGMA gene-property analysis is performed for gene expression of GWAS data (GTEx v8 30 general tissue types and 53 tissue types). TWAS, Transcriptome-Wide Association Study; MAGMA, Multi-marker Analysis of GenoMic Annotation; GWAS, Genome-Wide Association Study.

Next, we performed a Transcriptome-Wide Association Study (TWAS) using FUSION to identify gene-level associations with PSQ. We found 23 genes that passed multiple comparison correction (Supplementary Table 9; Figure 3b). Next, we performed a fine mapping analysis using FOCUS on genomic structural equation data, and 18 genes were found to be possible disease-causing signals for PSQ (pips>0.8; Supplementary Table 10). To further confirm these “highly credible” gene-level associations, we performed an intersection test. These include LMOD1, ZDHHC5, MAD1L1, MED19, etc. (Figure 3c). Among them, the TWAS Z-scores of LMOD1, ZDHHC5, etc., were all greater than 0, indicating that the predicted gene expression was positively correlated with PSQ, suggesting that upregulation of these genes may be associated with PSQ. In contrast, the TWAS Z-scores of MAD1L1, MED19, etc., were less than 0, indicating that downregulation of these genes may be associated with PSQ (Supplementary Table 11; Figure 4a).

### Pathways, cell types, and Mendelian genetic disease gene enrichment

**Figure 5:**
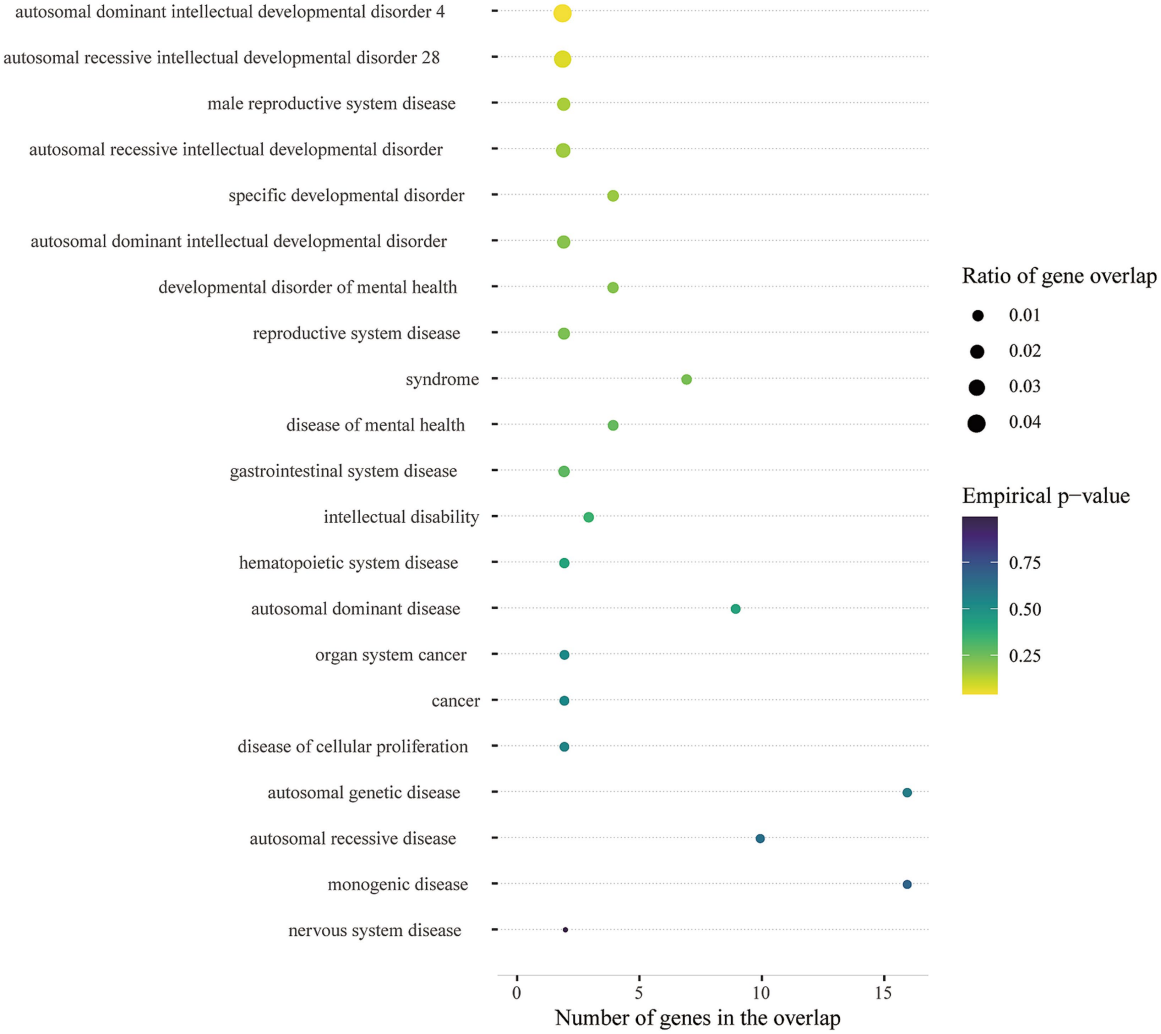
Disease ontology enrichment with MendelVar.

Multi-marker Analysis of GenoMic Annotation (MAGMA) identified 35 genes (bon-*p* < 0.05; Supplementary Table 12; Figure 4b), which we utilized for gene set analyses, and these genes showed enrichment in GSEA entries (Supplementary Table 13); many of the gene sets were associated with Glutamate synapse, *γ*-Aminobutyric acid synapse, Neuroticism, etc. In addition, biological processes mapped by MendelVar enrichment were supported by mapping in GSEA entries (autosomal dominant intellectual developmental disorder 4), however, there were no significant results after FDR correction (Figure 5). Analysis of enrichment from different cell types showed 5 cell types exceeding the significance criterion (Supplementary Table 14). Four of the 5 cell types were associated with the nervous system, namely Neuron, Oligodendrocyte, Oligodendrocyte precursor cell and Astrocyte. After FDR correction Neuron still exceeded the significance criterion (FDR-*p* < 0.05).

### Results of contribution of heritability on genomic regions

In the results of the contribution of heritability on genomic regions, we found that most of the genetically contributing sites are concentrated in the regulatory regions of chromosomes and histone modification regions such as H3K4me1. These regions are usually key sites for gene expression regulation, chromatin modification, and transcription factor binding. In particular, the effects of genetic variation are most pronounced in regions of conserved genomic regions and intronic regions, which may play an important role in traits or disease susceptibility by regulating gene expression levels. In addition, certain non-coding regions such as the 3’ Untranslated Region also showed strong genetic contributions, suggesting that these regions may be involved in complex genetic mechanisms by regulating gene expression or function (Supplementary Table 15).

### Biomarker and risk factor labeling analysis

**Figure 6:**
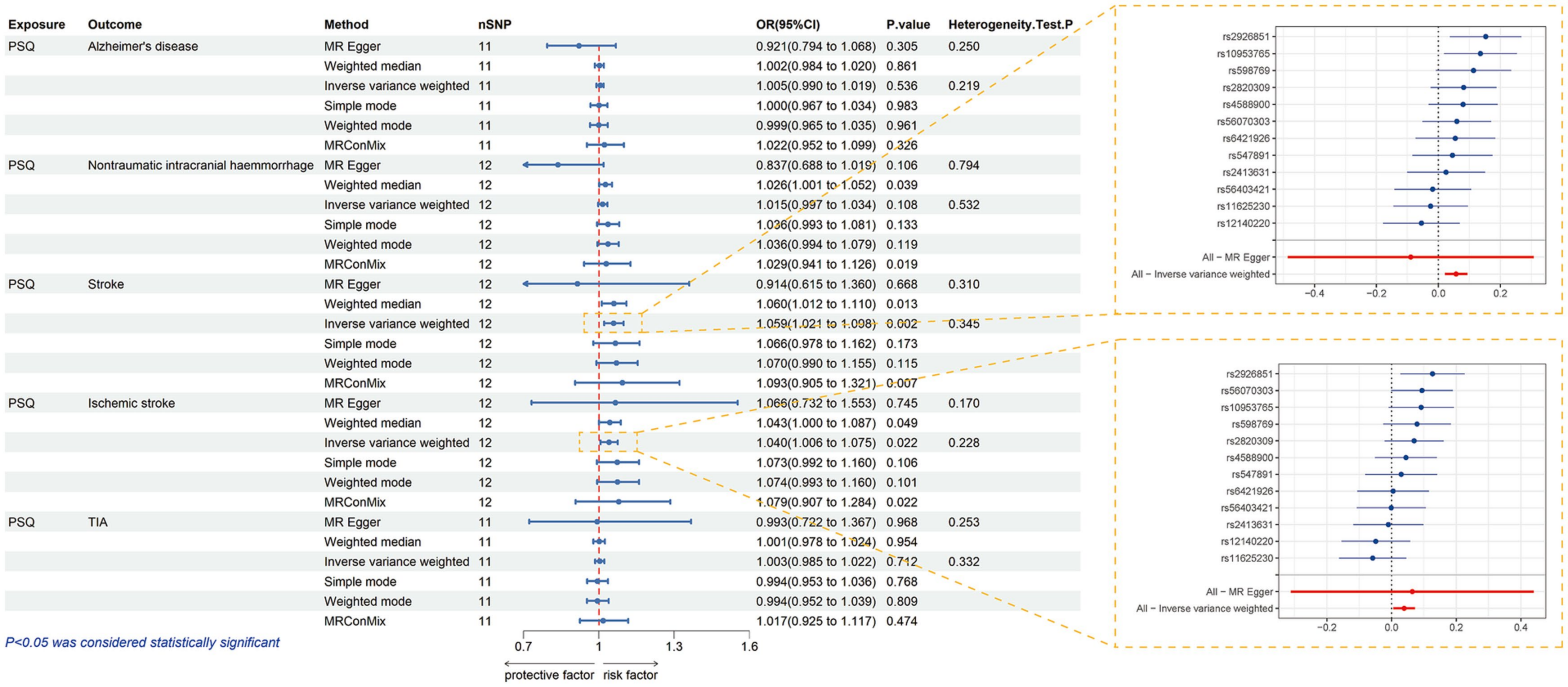
Forest plot of MR when PSQ is exposure and Alzheimer’s disease, non-traumatic intracranial hemorrhage, stroke, ischemic stroke, and TIA are outcomes, respectively; MR forest plot for stroke and ischemic stroke as outcomes. MR, Mendelian randomization; PSQ, poor sleep quality; TIA, transient ischemic attack.

In the results of biomarker analysis we found 705 positive exposures (Supplementary Table 16), in which genes such as PDK4 (ENSG00000004799) were positively correlated with PSQ, and on the contrary genes such as MAD1L1 (ENSG00000002822) were negatively correlated with it, and traits such as Daytime nap, Body mass index (BMI) may be risk factors for PSQ, while such as HDL cholesterol levels may be protective factors. After Bonferroni multiple correction, there are still 93 positive exposure factors. All of these factors may have a biological impact on sleep quality. In addition, we further investigated the potential relationship between PSQ and 20 common neurological disorders and found that PSQ was a risk factor for Stroke and Ischemic stroke, and all of them passed the tests of heterogeneity and multiplicity (Supplementary Table 17; Figure 6).

### Chromosome level results

Our analyses showed that PRS preformed variant loci were strongly associated with the risk of developing the disease and that the genetic contribution to the disease varied significantly among different chromosomal regions. In particular, we observed higher genetic contributions in regions such as chromosome 6 (−0.061) and chromosome 8 (−0.063), which may contain important genes and regulatory elements that influence disease susceptibility (Supplementary Table 18).

## Discussion

This study provides an in-depth exploration of the genetic basis of TFA, Ins, Und, SlD, HDD, and Tir, combining multiple methods such as Genomic-SEM, summary data PRS, MR, fine mapping and transcriptomics analysis. Through the joint analysis of these complex traits, we identified multiple new genetic markers. We found that genetic factors not only affect sleep quality, but may also have a profound impact on an individual’s entire life through the expression of cells, genes, risk factors, etc. This study provides a new theoretical basis for understanding how genetic loci shape PSQ and provides an important reference for the implementation of precision medicine and public health interventions in the future.

Our study revealed genetic covariates between single-input GWAS through Genomic-SEM analysis. The results suggest that shared genetic factors between these phenotypes, TFA, Ins and Und play an important role. The study suggests that TFA, and Ins in particular, may favor an anomalous overload state that impairs brain neuroplasticity and stress-immune pathways, leading to psychiatric disorders (Palagini et al., 2022). Secondly, the relationship between Ins and depression, psychiatric disorders, coronary heart disease, metabolic syndrome and hypertension has also been extensively studied. Studies have shown that treating Ins reduces the risk of developing cardiovascular and mental health disorders (Shaha, 2023). Regarding the relationship between Ins and hypertension, hypertension is affected by the duration of sleep. Human studies have shown that sleep deprivation (≤ 5 h/day) and Ins increase the risk of developing hypertension fivefold (Kwok et al., 2018; Gobbi and Comai, 2019). Multiple cross-sectional studies have shown a significant relationship between Ins and metabolic syndrome (MetS), and interventions that significantly improve sleep have the potential to positively impact MetS (Chasens et al., 2021). Each component of MetS is an independent risk factor for cardiometabolic disease (CVD), and the combination of these factors increases the incidence and severity of systemic inflammation and cardiovascular disease (Lopez-Candales et al., 2017). Taken together, SEM further confirms the complex genetic linkages between single-input GWAS, implying that these traits do not exist in isolation, but are intertwined and work together.

Through subsequent analysis of Genomic-SEM, multiple new SNPs were identified, which were significantly associated with traits such as cognitive function (rs6421926; Demange et al., 2021), insomnia (rs4588900; Watanabe et al., 2022), etc. Most of these newly discovered SNPs are located in intronic and intergenic regions, indicating that introns and non-coding regions may play an important role in genetic mechanisms. Previous studies have shown that introns can regulate gene expression by affecting RNA splicing, especially during the process of alternative splicing, introns can determine different splicing variants, thereby affecting protein diversity and function (Monteuuis et al., 2019). In addition, non-coding variants may play a role by affecting gene expression, rather than directly altering protein function (Tak and Farnham, 2015). Variants in non-coding regions may also affect gene expression by influencing the formation or disruption of transcription factor binding sites (Degtyareva et al., 2021). These sleep-related SNP studies provide potential genetic targets for follow-up studies and offer new insights into understanding the genetic links between sleep-related traits.

In this study, multiple key SNPs were identified through fine-tuned localization in gene regions associated with neurodevelopmental, mental health, and other disorders. These findings are consistent with previous findings (Cross-Disorder Group of the Psychiatric Genomics Consortium, 2019), who found that one study identified 109 loci associated with at least two psychiatric disorders through genomic association analysis of eight psychiatric disorders. This included 23 loci with pleiotropic effects on four or more disorders, suggesting that key loci for neurodevelopment and mental health are often highly associated with specific SNPs. The identification of these SNPs has enabled us to understand more precisely how these genes affect an individual’s health and disease susceptibility by influencing processes such as sleep quality and neurological changes. In particular, we have identified genetic markers in multiple regions of genes associated with the nervous system and sleep, suggesting that these regions may play a key role in the development of phenotypes with PSQ.

We further identified potential disease-causing genes associated with these SNPs through FUSION transcriptomics analysis and FOCUS fine localization analysis. Most of these genes are involved in important biological pathways such as intellectual development, visceral smooth muscle cell contraction, and lipid metabolism, and are closely associated with known disease-associated pathways such as central nervous system (CNS) disorders. Interaction between the CNS and the immune system plays an important role in regulating immune responses, and maintenance of this interaction is critical especially in neurodevelopment and adult plasticity (Tian et al., 2012). Additionally, studies have shown that neurological disorders may play a role by affecting the mechanisms that regulate contraction of visceral smooth muscle. The regulation of smooth muscle contraction involves complex signaling pathways, including calcium signaling and changes in actin cytoskeleton dynamics (Gunst and Zhang, 2008; Sanders, 2008). Finally, the role of lipids in neurological disorders cannot be ignored. Lipids play a key role in the physiology and pathology of CNS cells, and their metabolic disorders may lead to serious neurological disorders (Ngo, 2021). These pathways may play an important role in the genetic basis of the PSQ phenotype.

Through MR analysis, we identified multiple known risk factors that are closely related to the occurrence of the disease. Some risk factors are related to lifestyle and environmental factors, such as smoking, eating habits, and physical activity, which is consistent with previous research results (Gheisary et al., 2024; Flor-Alemany et al., 2020; Zheng et al., 2024). The interaction between genes and the environment plays an important role in the pathogenesis of complex diseases. Studies have shown that the genetic effects of individuals may be modified by environmental factors at the group level, such as the living environment. This interaction can be quantitatively analyzed using Bayesian models (Wang et al., 2013). Future research should focus more on how to combine genetic and environmental factors and consider how these factors work together to promote sleep quality and further research into the nervous system. The study of gene–environment interactions not only helps us better understand the pathogenesis of complex diseases, but also provides a theoretical basis for the development of personalized prevention and intervention strategies. Consistent with evidence from other sleep-related traits, obstructive sleep apnea (OSA)—a clinically defined sleep disorder—shows genetically predicted causal associations with both brain structure and cognitive performance in Mendelian randomization analyses (Bao et al., 2024). Such findings illustrate that genetic liability for sleep-related conditions can have downstream effects on neural morphology and cognitive function, highlighting potential pathways by which sleep-related traits may interact with psychosocial and metabolic factors in shaping health outcomes. In addition, we have identified several novel genetic susceptibility factors that may increase susceptibility to disease by influencing physiological processes or gene expression such as lipid metabolism and blood pressure regulation. These risk factors provide new directions for further clinical research and drug target development. Finally, we also found that PSQ may cause acute CNS damage such as stroke. Previous studies have mainly focused on the impact of PSQ after stroke (Rangel et al., 2024; Babkair et al., 2023) and traumatic brain injury (TBI) on the recovery process (Johnson et al., 2019). Our study provides a new perspective on the prevention of acute cerebrovascular diseases such as stroke.

By analyzing genome-wide data, we identified multiple risk chromosomal regions associated with PSQ. These regions affect various biological processes by regulating the expression of nearby genes. For example, we identified multiple risk loci on chromosomes 1, 5, 8, 11, 14 and 18 that are associated with sleep, neurological disease, and other traits. These loci are often enriched in functional gene regulatory regions (such as enhancers and promoters) and intronic regions. These genomic elements may play a key role in the genetic background of the disease. Studies have shown that errors in intron splicing can lead to abnormal gene expression, which can trigger disease (Borišek et al., 2021). In addition, studies of genome enhancer maps have revealed how risk variants affect disease genes through enhancers in different cell types (Nasser et al., 2021). In a broader context, the role of non-coding genetic variants in a variety of complex diseases has also been studied. Studies have shown that non-coding variants may affect disease risk by influencing chromatin states and gene regulatory networks (Chawla et al., 2021; Grubert et al., 2015). For complex diseases such as sleep disorders and the nervous system, studies have already shown that these genomic regulatory regions are closely related to the expression of disease-related genes (Konki et al., 2019; Zhang et al., 2024). Finally, we observed a higher genetic contribution in regions such as chromosomes 6 and 8, which may contain important genes and regulatory elements that affect disease susceptibility.

Although this study provides new insights into the genetics of PSQ, there are still some limitations. First, the sample population in this study was mainly of European descent, and validation in other populations is lacking. Therefore, future studies should expand the sample population and validate the findings in particular in populations of different races and regions to ensure the broad applicability of these genetic findings. Second, although our multivariate analysis method has improved statistical power, the low h^2^ value and possible residual phenotypic heterogeneity suggest that these findings should be interpreted with caution and may require verification in datasets with more precise sleep phenotypes. In addition, although we have identified multiple genetic loci associated with PSQ through fine mapping and transcriptomics analysis, how to link these genes to specific biological mechanisms remains an urgent problem. Future research needs to explore in depth the mechanisms by which these genetic variants affect sleep quality and neurological diseases. Finally, although our research has revealed the important role of genetic factors in PSQ, the role of environmental factors should not be ignored. Future research should further explore how environmental factors (such as eating habits, living environment, smoking, and life stress) affect the expression of these phenotypes through interactions with genetic factors, and promote in-depth research on gene–environment interactions.

This study provides new insights into the genetic basis of poor sleep quality. By genomic structural equation modeling, we identified multiple novel genetic loci and revealed how these loci affect the genetic link between gene expression and complex traits. Our findings deepen the understanding of the biological mechanisms underlying poor sleep quality. However, a cautious interpretation is warranted, and replication in independent datasets, together with formal colocalization and functional validation, will be essential for determining the translational relevance of these genetic findings. While these results may eventually contribute to precision medicine and public health strategies, further work is required to substantiate their clinical utility. Future studies will also explore the role of gene–environment interactions in sleep and neurological disorders, with the aim of improving healthy lifespan and quality of life worldwide.

## Data Availability

Publicly available datasets were analyzed in this study. This data can be found at: IEU OpenGWAS: https://gwas.mrcieu.ac.uk and FinnGen: https://www.finngen.fi/en. Other websites used are listed at: MendelVar https://mendelvar.mrcieu.ac.uk/submit/.

## Footnotes

1 https://mendelvar.mrcieu.ac.uk/submit/
